# Valuable effect of Manuka Honey in increasing the printability and chondrogenic potential of a naturally derived bioink

**DOI:** 10.1016/j.mtbio.2022.100287

**Published:** 2022-05-13

**Authors:** Annachiara Scalzone, Giorgia Cerqueni, Maria A. Bonifacio, Michele Pistillo, Stefania Cometa, Monica Mattioli Belmonte, Xiao N. Wang, Kenny Dalgarno, Ana M. Ferreira, Elvira De Giglio, Piergiorgio Gentile

**Affiliations:** a School of Engineering, Newcastle University, Newcastle upon Tyne, United Kingdom; b Department of Clinical and Molecular Sciences (DISCLIMO), Università Politecnica delle Marche, Ancona, Italy; c Department of Chemistry, University of Bari “Aldo Moro”, Bari, Italy; d INSTM, National Consortium of Materials Science and Technology, Florence, Italy; e Jaber srl, Rome, Italy; f Translational and Clinical Research Institute, Newcastle University, Newcastle upon Tyne, United Kingdom

**Keywords:** Methacrylated gellan gum, Manuka honey, Mesenchymal stem cells, Extrusion bioprinting, Articular cartilage

## Abstract

Hydrogel-based bioinks are the main formulations used for Articular Cartilage (AC) regeneration due to their similarity to chondral tissue in terms of morphological and mechanical properties. However, the main challenge is to design and formulate bioinks able to allow reproducible additive manufacturing and fulfil the biological needs for the required tissue. In our work, we investigated an innovative Manuka honey (MH)-loaded photocurable gellan gum methacrylated (GGMA) bioink, encapsulating mesenchymal stem cells differentiated in chondrocytes (MSCs-C), to generate 3D bioprinted construct for AC studies. We demonstrated the beneficial effect of MH incorporation on the bioink printability, leading to the obtainment of a more homogenous filament extrusion and therefore a better printing resolution. Also, GGMA-MH formulation showed higher viscoelastic properties, presenting complex modulus G∗ values of ∼1042 ​Pa, compared to ∼730 ​Pa of GGMA. Finally, MH-enriched bioink induced a higher expression of chondrogenic markers *col2a1* (14-fold), *sox9* (3-fold) and *acan* (4-fold) and AC ECM main element production (proteoglycans and collagen).

## Graphical abstract

**Figure:**
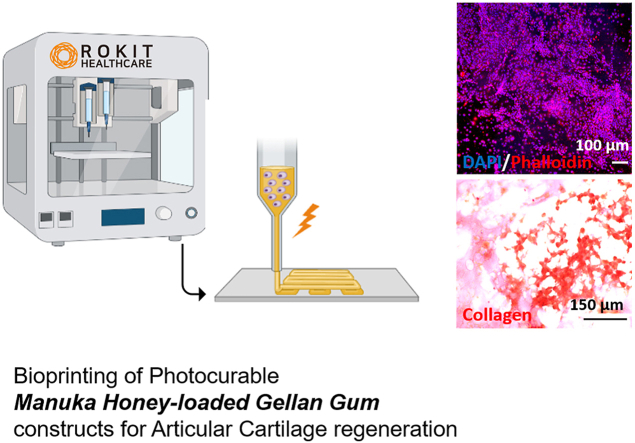

## 1 Introduction

Articular cartilage (AC) is a highly specialised connective tissue of diarthrodial joints [1]. Its damages are commonly caused by trauma or inflammation; however, the injured cartilage has a limited capacity for intrinsic regeneration, due to its avascular, aneural and alymphatic nature [2]. Hence, if not treated, the damage often develops into degenerative arthritis and may lead to the loss of entire joint function. This issue has driven keen endeavours in orthopaedic research over the last few decades [3]. Currently, the standard clinical treatment for joint degeneration is a total joint replacement using metallic prostheses, which lack biologically adaptive properties and thus, have a limited life span. Other clinical therapies, e.g. microfracture or autologous chondrocyte implantation, fulfil pain relief for the patient and short-term treatments, but are often unable to restore the long-lasting healthy cartilage [4]. In this scenario, the introduction of AC *in vitro* models would advance the research into novel therapeutic treatment for damaged and diseased AC, while supporting the 3R's philosophy based on the reduction of animal testing for research purposes [5]. One of the main requirements for obtaining a reliable *in vitro* model, is to engineer constructs able to biomimic the properties of AC tissue. In this regard, three-dimensional bioprinting technology, which is radically changing regenerative medicine, is allowing the obtainment of tissue-engineered constructs with appropriate control over spatial variations, the capability of precise deposition of cells, biomaterials, growth factors, and other bioactive reagents to build cell-laden constructs [[6], [7], [8]]. Particularly, extrusion-based bioprinting is the most widely exploited because it is simple, user-friendly, and allows the fabrication of scalable and structurally stable constructs with high cell density (>5x10^6^ ​cells/mL) in a relatively short time [9,10].

However, one of the challenges of bioprinting is to identify bioinks that, simultaneously, fulfil the requirements for reproducible additive manufacturing as well as the biological needs for the cells of the tissue aimed to replace, by supporting cell growth and differentiation, tissue maturation and, ultimately, the formation of a functional construct. An ideal bioink material should meet the following requirements: (i) printability, (ii) high mechanical stability, (iii) insolubility in the culture medium, (iv) cytocompatibility and non-immunogenicity, (v) quickly production and commercial feasibility, and (vi) cell viability, proliferation, and biosynthetic activity promotion [11]. Bioprinting of decellularized ECM [[12], [13], [14]] and of several natural polymers for AC applications has been reported for hyaluronic acid (HA), gelatin (Gel), gellan gum (GG), chitosan, agarose, collagen and alginate [[15], [16], [17], [18], [19]]. However, due to unsuitable mechanical properties and the inability to be self-supporting for multi-layered fabrication, the printing fidelity is very limited, and it is always difficult to produce large-scale functional tissue constructs [20]. Particularly, methacrylate-containing materials are commonly used with hydrogels for cartilage regeneration [[21], [22]]. Among these, thermo- and photo-responsive gelatin methacrylamide (GelMA) has been proven as a versatile and promising platform for AC tissue engineering; however, the mechanical properties of the obtained constructs are still questionable [23]. Among natural-based biomaterials, gellan gum (GG), which has FDA approval as a food additive, has temperature-responsive gelation property, biocompatibility, and low toxicity, thus gains increasing attention in AC tissue engineering (TE) [24]. Although GG has been exploited for different clinical needs (e.g. mucoadhesive excipients for ocular drug delivery [25], cardiac TE and disease modelling [26], bone and cartilage TE applications [27,28], GG-based hydrogels are usually produced only by means of ionically crosslinking mechanisms in cationic solutions, yielding hydrogels that become weaker in physiological conditions due to the exchange of divalent cations by monovalent ones, as described elsewhere [29]. Therefore, a good way for increasing its thermal stability is the introduction of methacrylate groups into GG polymeric chains via chemical modification, in order to better control the gelation process after UV exposure and in the presence of a photoinitiator [30].

Thus, we decided to investigate the potential of photocurable bioprinted methacrylated GG (GGMA) hydrogels. GGMA hydrogels have been already exploited for intervertebral disc tissue-engineering applications, showing good porosity, viscoelastic properties and cytocompatibility. These properties are fundamental for our application as a biomimetic AC-like *in vitro* model [31]. Furthermore, methacrylated GG can be ionically crosslinked with the supply of cations present in cell culture medium, which enable the aggregation of GG helical domains [32]. Herein, we introduced the medical grade Manuka honey (MH), derived from New Zealand *Leptospermum scoparium*, for preparing GG-based bioinks, since we have already demonstrated MH intrinsic antibacterial activity and enhanced mechanical properties, suitable for AC tissue-engineering, when added to GG-based hydrogels [[33], [34], [35], [36]].

This work aimed to propose an innovative bioink suitable for extrusion-based bioprinting technology and to generate an *in vitro* biomimetic AC-like tissue model to be used to further test therapeutic compounds. To this aim, GG was methacrylated and the physicochemical and mechanical properties of the developed hydrogels, with and without MH addition, were investigated. Then, GGMA-based hydrogels were used as bioinks, and the effect of the incorporation of MH on printability, rheological properties, cell viability and chondrogenic behaviour were evaluated. For biological studies, to reduce the variability that primary cell cultures can generate and achieve high cell yield, chondrocytes differentiated from immortalised mesenchymal stem cells (MSCs-C) were used [37]. Finally, this study explored the feasibility of obtaining a cartilage-like bioprinted construct by evaluating the chondrogenic attitude of MSCs-C within cells-laden MH-enriched naturally derived GGMA-based bioinks *in vitro* via gene expression analysis, histology, and glycosaminoglycans quantification.

## 2 Materials and methods

### 2.1 Materials

All materials were bought from Sigma Aldrich UK, unless otherwise stated. The medical grade Manuka Honey (MGO 400) was supplied by ManukaGuard (US).

### 2.2 GGMA and GGMA-MH hydrogels preparation

The synthesis of the GGMA by reacting Gellan Gum (Gelrite®, Molecular weight (Mw) ​= ​1.000.000 ​Da) with methacrylic anhydride (MA) and re-adapting a previous protocol by Coutinho et al. [29] is reported in the supplementary data. For the GGMA and GGMA-MH hydrogels preparation, freeze-dried GGMA was sterilised under UV (254 ​nm) and then dissolved at 2% (w/v) in dH_2_O under constant stirring at 50°C for 3–4 ​h covered from light. This solution was supplied with the addition of the lithium phenyl 2,4,6-trimethylbenzoyl-phosphinate (LAP) photoinitiator (0.1% w/v). For the Manuka Honey-loaded formulation, the MH was added to the GGMA solution at a concentration of 5% w/v, at the start of the dissolution process. When a complete and homogeneous dispersion of the material was obtained, 1 ​mL of GGMA and GGMA-MH solutions were poured in bijou vials and photo-crosslinked by exposing to light (365 ​nm, 8 ​W/m^2^ supplied by Rokit INVIVO Bioprinter) for up to 10 ​min and ionically crosslinked with the addition of an equal volume of cell culture medium (Dulbecco's Modified Eagle Medium/Nutrient Mixture F-12, DMEM/F12, ThermoFisher, UK) with divalent ions. The hydrogels obtained were stored in the incubator at 37°C and 5% CO_2_. Some of the obtained hydrogels were stored at −20°C overnight and then lyophilised for 48 ​h in a freeze-dryer, as reported before (section 2.2).

### 2.3 Hydrogels characterization

#### 2.3.1 Gelation time

Hydrogels’ gelation time was measured at room temperature (RT) using the test tube inversion method [40]. GGMA and GGMA-MH solutions (1 ​mL) were placed under UV light to measure the gelation time. The test was performed on a bare solution and on a solution with the addition of cell culture medium (500 ​μL) after 1 ​min of exposition to UV light. The sample flowability was observed every 30 ​s, by tilting the vials, considering the gelation time when the flow of the solution stopped.

### 2.4 Water uptake kinetics

To study the water uptake ability of the developed hydrogels, three freeze-dried samples for each composition were used. GGMA and GGMA-MH lyophilised gels (5 ​mm diameter x 6 ​mm height) were weighted singularly and immersed in 3 ​mL of Dulbecco's phosphate buffered saline (PBS) in a 5 ​mL bijou vial and stored at 37°C. At different time points (30 ​min, 1, 3, 5, 8, 24 and 48 ​h of incubation), the hydrogels were removed from the solutions and gel surfaces were quickly blotted on a filter paper. Their wet weight was measured (Wt) and compared to the initial wet weight (Wi). The water uptake (WU) was defined according to Eq [1].:

(1) $$WU\left(\%\right)=\frac{W_{t}-W_{i}}{W_{i}}\;\cdot \;100$$

### 2.5 Morphological analysis

Freeze-dried hydrogels morphology was investigated by JEOL JSM-5600LV Scanning Electron Microscope. Samples were cut into small squares (2 ​mm diameter x 1 ​mm height), fixed on the aluminium stub using carbon tape and gold-coated using a BIO-RAD Sputter Coater machine. Thus, these were finally analysed at a 6 ​mm working distance, a 20 ​kV operation voltage used at different magnifications (35x and 100x). The pictures were analysed through ImageJ software, to evaluate the frequency of pores diameter distribution for each sample. Three images for sample type were analysed measuring 50 pores for each one. The pore size was averaged to give a mean pore size assuming all pores were circular.

### 2.6 Mechanical characterization

Unconfined compression tests were performed using a mechanical testing machine (EZ-SX, Shimadzu, Japan) equipped with 20 ​N loading cell, on freshly prepared GGMA and GGMA-MH hydrogels (6 ​mm diameter x 5 ​mm height). Three samples for composition were compressed in the direction normal to the circular face of the cylindrical samples at a rate of 1 ​mm.min^−1^ until failure of the hydrogel (∼30% for GGMA and 60% for GGMA-MH of original height). A stress/strain (σ/*ε*) graph was plotted. From this graph, Young's modulus (E) was calculated as the slope of the linear-elastic region of the σ/*ε* curve (0–10% strain).

Rheological analyses were performed with Kinexus Pro rheometer (Malvern Instruments Ltd, UK) using a cone and plate geometry with a 2 ​mm gap. The temperature of the samples was controlled with an accuracy of ±0.1°C, by Peltier system of the rheometer. Measured data were registered with rSpace for Kinexus Pro 1.3 software. The Temperature Sweep Test was carried out to assess the hydrogels' behaviour at different temperatures, setting a temperature increase at a rate of 5°C in the range of 15–50°C and the values of G′ and G″ were recorded in the Linear Viscoelastic Region (LVER) for each temperature. The oscillatory measurement was set at a frequency of 1 ​Hz and 1% strain amplitude. The Strain Sweep Test was performed at physiological temperature of 37°C to verify the values of the strain amplitude to identify the LVER and to assess the material's stretchability. This test was performed with a rotational oscillation frequency of 1 ​Hz. The complex modulus G∗, was recorded with the following equation (Eq. (2)):

(2) $$G^{\text{∗}}=\sqrt{{\left(G^{\text{′}}\right)}^{2}+{\left(G"\right)}^{2}}$$

Also, the apparent viscosity (η) and the strain value at yield point were recorded.

### 2.7 Cells culture

Human TERT immortalised bone marrow stromal cell line was cultured and differentiated into chondrocytes (MSCs-C) as reported before [38]. Briefly, cells were grown at 37°C, 5% CO_2_ in DMEM/F12 supplemented with 10% Fetal bovine serum (FBS), 2 ​mM l-glutamine and a 1% Penicillin/Streptomycin (P/S). When 80% confluence was reached, cells were differentiated in chondrocytes, using a mixture of chondrogenic factors (serum-free DMEM with P/S supplemented with 1% ITS+1, 10 ​ng/mL TGF-β3, 40 ​μg/mL l-Proline, 100 ​nM Dexamethasone, 50 ​μg/mL l-Ascorbic acid-2-phosphate) for 21 days. MSCs-C were cultured in DMEM/F12 supplemented with 10% FBS and 1% P/S and used at passage 15 after differentiation.

### 2.8 Bioinks fabrication and 3D bioprinting

MSCs-C cells (passage 15) were detached using trypsin EDTA, centrifuged at 1200 ​rpm for 5 ​min and resuspended in the GGMA or GGMA-MH solutions at a concentration of 7 ​× ​10^6^ ​cells/mL. These bioinks were then transferred to the printing cartridge of a Rokit INVIVO 3D Bioprinter (Rokit Healthcare), equipped with a bio-dispenser to extrude bioinks in a sterile environment (HEPA filters) with controlled temperature. The temperature of the bio-dispenser was set to 36°C, while the one of the printing-bed to 25°C, as the viscosity of the bioinks increased at RT compared to physiological conditions. Before the printing, the x, y and z calibrations were done manually and a multi-layered squared grid structure (6 ​mm ​× ​6 ​mm x 2 ​mm) was realized using the NewCreatorK software (Rokit Healthcare). A 27 ​G tip was chosen to print the bioinks with a speed of 5 ​mm/s. The UV-led (wavelength 365 ​nm) available below the printhead was switched on manually to permit photo-polymerization during the printing process (3 ​min per sample). The constructs were printed on a layer of sterile DMEM/F12 upon a glass Petri dish (diameter 9 ​cm) to allow physical crosslinking. Then, samples were transferred to 48-well plates containing 500 ​μL of DMEM/F12 supplemented with 10% FBS and 1% P/S and stored in the incubator for up to 21 days for further analysis, with a change of media every two days. Printability was evaluated for both formulations by analysing the filament spreading ratios, calculated as the width of the printed filament divided by the needle diameter, and the filament diameter after the deposition post-crosslinking on images taken with an EVOS M500 microscope. Images were processed with Image J software. Triplicates of samples were printed for each time point for the biological studies.

### 2.9 Evaluation of cell viability, metabolic activity, and morphology

Cell viability assays were performed to evaluate the impact of the bioprinting process on cell behaviour and the cytocompatibility of the photocrosslinked hydrogels. For the GGMA and GGMA-MH constructs, a ReadyProbes® Cell Viability Imaging Kit (Thermo Fisher, UK) was used. Thus, NucBlue® Live reagent (Hoechst 33342), staining the nuclei of all the cells (blue) was combined with ethidium bromide to obtain viability information by comparing total vs dead cells. After 1 and 3 days from the bioprinting process, the medium (DMEM/F12 ​+ ​FBS ​+ ​P/S) was removed from each sample. The samples were washed with PBS and then incubated for 30 ​min at 37°C with the staining solution: 4 ​μL ethidium homodimer-1 and 4 drops of Hoechst in 2 ​mL of PBS. Images were collected on day 1 and day 3 using a Nikon A1R inverted confocal microscope. The number of dead cells was evaluated and reported in Supplementary data.

MTS(3-(4,5-dimethylthiazol-2-yl)-5-(3 Carboxymethoxy-phenyl)-2- (4-sulfophenyl-2H-tetrazolium) assay (CellTiter 96® AQueous One Solution Cell Proliferation Assay, Promega, UK) was used to assess cells metabolic activity at days 1, 3 and 7 of constructs culture. MTS solution was prepared with CellTiter 96® AQueous One Solution Reagent in phenol red-free DMEM/F12 supplemented with 10% FBS and 1% P/S (1:5). 200 ​μL of MTS solution was added to the samples, incubated at 37°C in a humidified 5% CO_2_ atmosphere for 2.5 ​h and then 90 ​μL of the solution were transferred into a 96-well plate in duplicate for each sample. Absorbance was recorded at 490 ​nm using a FLUOstar® Omega multi-mode reader and compared with the value of a known cellular number.

For fluorescent labelling, samples on days 1, 3 and 7 were fixed in pre-warmed 4% w/v paraformaldehyde (PFA) for 30 ​min at 4°C, after being washed in PBS. Then, cells were re-washed in PBS and permeabilised using 0.1% v/v Tween20® in PBS for three washes and then samples were incubated with phalloidin-tetramethylrhodamine B isothiocyanate (Phalloidin Rhodamine) solution (1:1000 in 0.1% PBS/Tween20®) for 30 ​min at RT. Following, samples were washed with 0.1% PBS/Tween20® solution and immersed in 4′,6-diamidino-2-phenylindole (DAPI) solution (Vector Laboratories, UK) (1:2500 in 0.1% PBS/Tween20®) for 10 ​min at RT. Then, images were collected using a Nikon A1R inverted confocal microscope and analysed with NIS-Elements Microscope Imaging Software.

### 2.10 Histological analysis

On day 21 samples were fixed in 10% Formalin solution overnight at 4°C and then embedded in a solution of OCT compound (Agar Scientific Ltd) and PBS (1:1) overnight at RT. Finally, samples were embedded in OCT compound within histological cryomolds and stored at −80°C. Following this, samples were cryosectioned in 5 ​μm-thick slices with a Cryostat (Leica). The slices were fixed on polylysine charged glass slides and stored at −80°C. The staining with Haematoxylin & Eosin (H&E), Alcian Blue and PicroSirius Red was performed following the manufacturer's instruction [40,42] After the staining, slides underwent a dehydration process in three rapid changes of absolute alcohol, then cleared in Histoclear solution (Scientific Laboratory Supplies) and mounted in DPX (Millipore, USA) mounting for histology. Stained slides were covered with a glass slide and imaged with an EVOS M5000 microscope.

### 2.11 Glycosaminoglycans quantification

Quantitative assessment of GAGs production in both GGMA and GGMA-MH samples was performed with Alcian Blue (pH 2.5) staining on days 1, 7 and 21 of culture. At each time point, samples were fixed with 4% PFA as explained before and then washed twice with PBS. Following, 500 ​μL of Alcian Blue solution were added to each sample for 30 ​min and then, samples were washed with dH_2_O until the complete removal of non-binded stain. After, 28.66 ​g of guanidine hydrochloride were dissolved in 50 ​mL of dH_2_O and 500 ​μL of the obtained guanidine solution were added to each sample and let for 3 ​h while shaking. Then, 100 ​μL were taken in triplicate from each well and reading was performed in absorbance at 630 ​nm with a Filter-based multi-mode microplate reader in a 96-clear bottom well plate. Results were reported for both bioinks considering a calibration curve obtained from the bare chondroitin 4-sulfate sodium salt from bovine trachea in a range of 0–1 ​μg.

### 2.12 Quantitative real time PCR analysis

At day 21 samples were washed with PBS and frozen at −80°C. Reverse Transcription Quantitative Real Time PCR (RT-qRT) was performed as explained previously [38]. Briefly, RNA was isolated using miRNeasy Micro RNA Isolation Kit (Qiagen, USA) and its concentration was measured using a spectrophotometer (NanoDrop™ 1000, Thermo Fisher Scientific, US). The cDNA retro-transcription was performed with the High-Capacity cDNA Reverse Transcription Kit (ThermoFisher, UK) in a thermocycler (2720 Thermal Cycler, Applied Biosystems, US) based on cycles of 25°C (10 ​min), 37°C (120 ​min), 85°C (5 ​min). RT-qPCR was performed using TaqMan™ Fast Advanced Master Mix and commercially available TaqMan qRT-PCR probes: SOX9 (Hs00165814_m1), ACAN (Hs00153936_m1), COL2A1 (Hs00264051_m1) and GAPDH (Hs99999905_m1) (ThermoFisher Scientific, UK) in a RT-qPCR analyser (QuantStudio 3, Thermo Fisher Scientific, US). The gene expression results were normalized to GAPDH and relatively quantified using the ΔΔCt method of Livak [39]. The expression levels of GGMA-MH samples at day 21 were calculated as fold change, with the expression levels of GGMA samples at day 21 as calibrator.

### 2.13 Morphological analysis of cells-laden bioprinted constructs

Cell morphology within the GGMA and GGMA-MH constructs was observed using a SEM (Tescan Vega LMU SEM) after 21 days of culture. Samples were fixed in pre-warmed 2% Glutaraldehyde overnight, rinsed in 0.5 ​M cacodylic acid buffer and dehydrated in ethanol grades: 30 ​min in each 25%, 50%, 70%, 80%, twice in 95% and four times in 100% EtOH. Samples were stored at 4°C in 100% EtOH until critical point dried using hexamethyldisilazane (HMDS). Finally, gels were mounted on carbon discs (TAAB Laboratory Equipment) and gold-coated using a Polaron E5000 SEM Coating unit (Quorum Technologies Ltd, UK). After gold coating, samples were imaged at different magnifications.

### 2.14 Statistical analysis

The statistical significance of the obtained results was evaluated by GraphPad Prism Software (v. 8.4.1), using One-way ANOVA with repeated measurements. Then, Tukey's post hoc test was carried out to highlight the main factors determining data variability. Statistical significance was set at ∗ p< ​0.05, ∗∗ p ​< ​0.01, ∗∗∗ p< ​0.001 and ∗∗∗∗ p< ​0.0001.

## 3 Results

### 3.1 Physico-chemical and mechanical properties of GGMA and GGMA-MH gel formulations

**Fig. 1:**
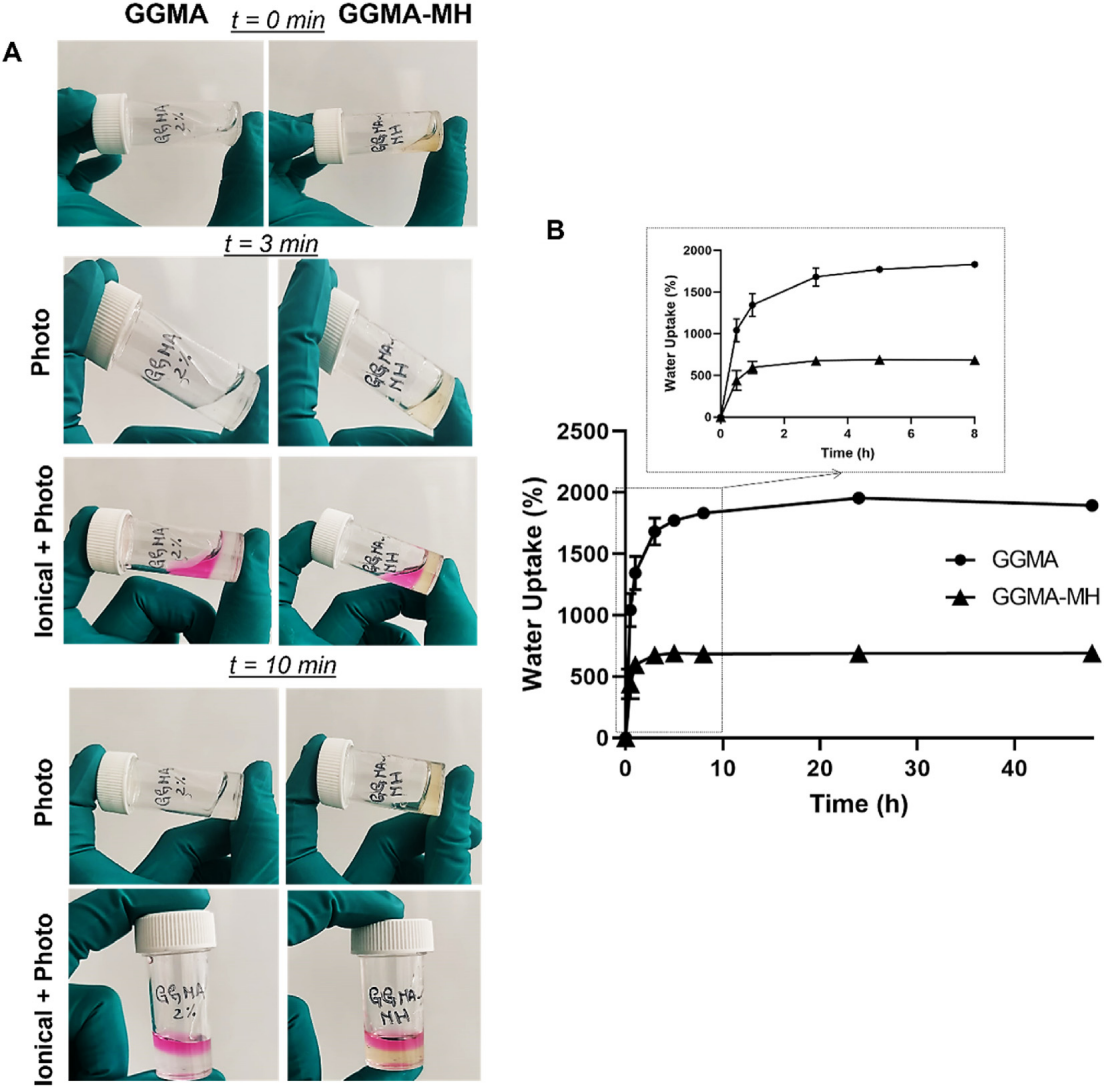
(**A**) Gelation time for GGMA and GGMA-MH hydrogels with photocuring or a combination of ionical- and photo-crosslinking: Tube inverted vial test at 0, 3 and 10 ​min; (**B**) Water Uptake study of GGMA and GGMA-MH at different time points (insert: zoom on the first 8h of uptake). Tests were performed in triplicates.

The chemical characterization of the synthesized GGMA raw materials is reported in the supplementary data. GGMA (2% w/v) and GGMA-MH (2% w/v- 5% w/v) solutions were prepared and 1 ​mL of each was poured into a bijou vial and placed under the UV light, to assess the gelation time with the tube inversion methodology. The experiment was performed under two different conditions: exposure of the solution to UV light to assess the gelation time related to photocrosslinking, which resulted to last 10 ​min for both GGMA and GGMA-MH; the second condition evaluated was the combination of photo and ionic crosslinking mechanisms, due to the presence of divalent ions in DMEM/F12 ​cell media, that was added to the GGMA and GGMA-MH solutions after 1 ​min of exposure to UV light. The dual crosslinking contributes to speed-up the crosslinking process, up to approximately 3 ​min for both formulations (Fig. 1A).

Fig. 1 B shows the water uptake ability of the GG-based hydrogel soaked in PBS at 37°C. GGMA samples displayed an initial rapid water uptake of 1400 ​± ​100% within 1 ​h. Then, the water uptake increased slowly over time, reaching a value of about 1800 ​± ​20% at 8 ​h and stabilised on this value up to 48 ​h. GGMA-MH samples instead had a fast-initial water uptake reaching a value of 500 ​± ​60% within 60 ​min and stabilised at this value up to 48 ​h.

**Fig. 2:**
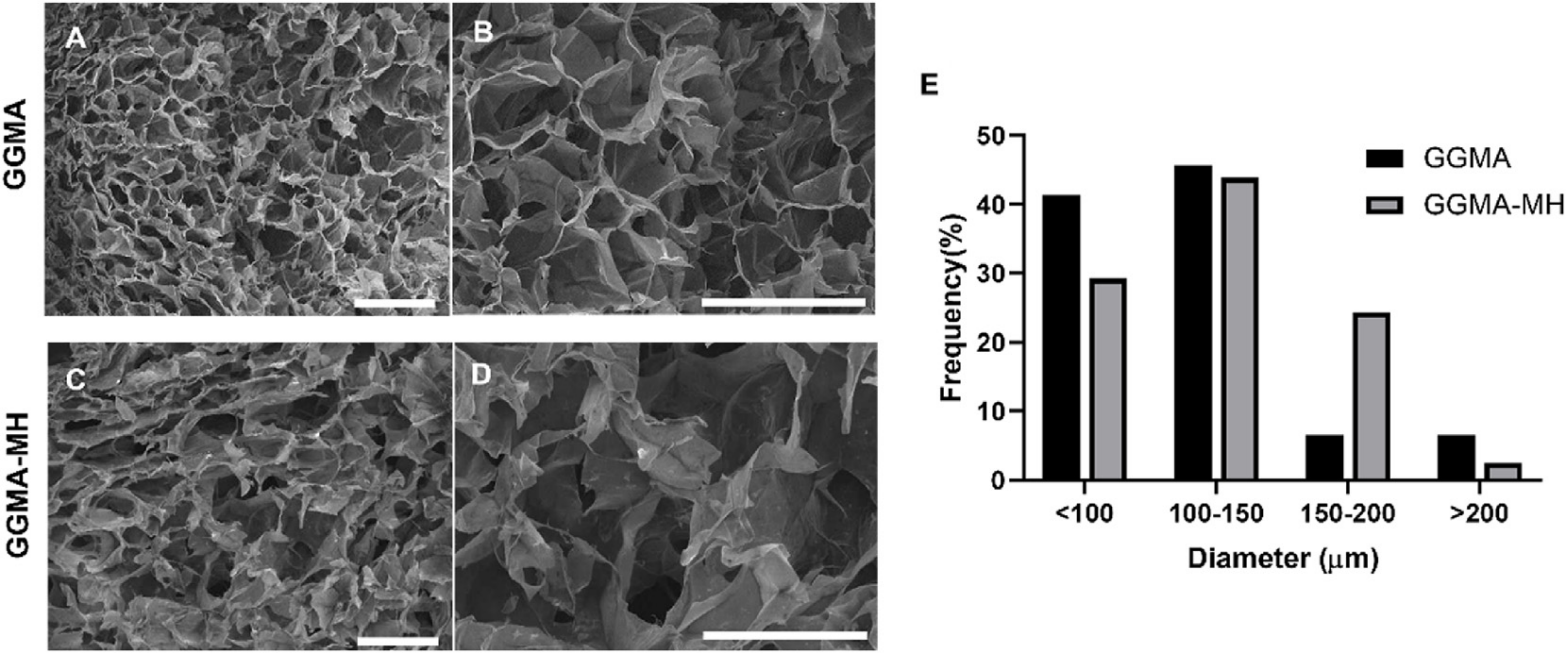
SEM images, representing cross-section microstructure of **(A,B)** GGMA and **(C,D)** GGMA-MH hydrogels at magnifications 35x **(A,C)** and 100x **(B,D)**. Bars ​= ​500 ​μm; **(E)** Frequency of pores diameter within the ranges: <100 ​μm, 100–1500 ​μm, 150–200 ​μm, >200 ​μm for GGMA (black) and GGMA-MH (grey). Tests were performed in triplicates.

From images of typical cross-sections of the freeze-dried GGMA and GGMA-MH hydrogels, it is possible to observe that both samples possessed pores with a typical spongy three-dimensional morphology, with open macropores, high degree of interconnectivity and anisotropic porosity (Fig. 2A–D). No differences were found in the morphological structure of the interior of all GG-based hydrogels analysed, although differences in the pores dimension distribution were observed. GGMA samples (Fig. 2E) presented 88% of pores with a diameter <150 ​μm, including 41% of pores with a diameter below 100 ​μm with an average pore size measured of 77.4 ​± ​17.6 ​μm and 47% of pores with a dimension in the range of 100–150 ​μm; while GGMA-MH samples presented the 73% of pores with a diameter <150 ​μm, most of them in the range 100–150 ​μm (44%) and 29% with a diameter <100 ​μm, with an average pore size measured of 87.2 ​± ​9.5 ​μm.

**Fig. 3:**
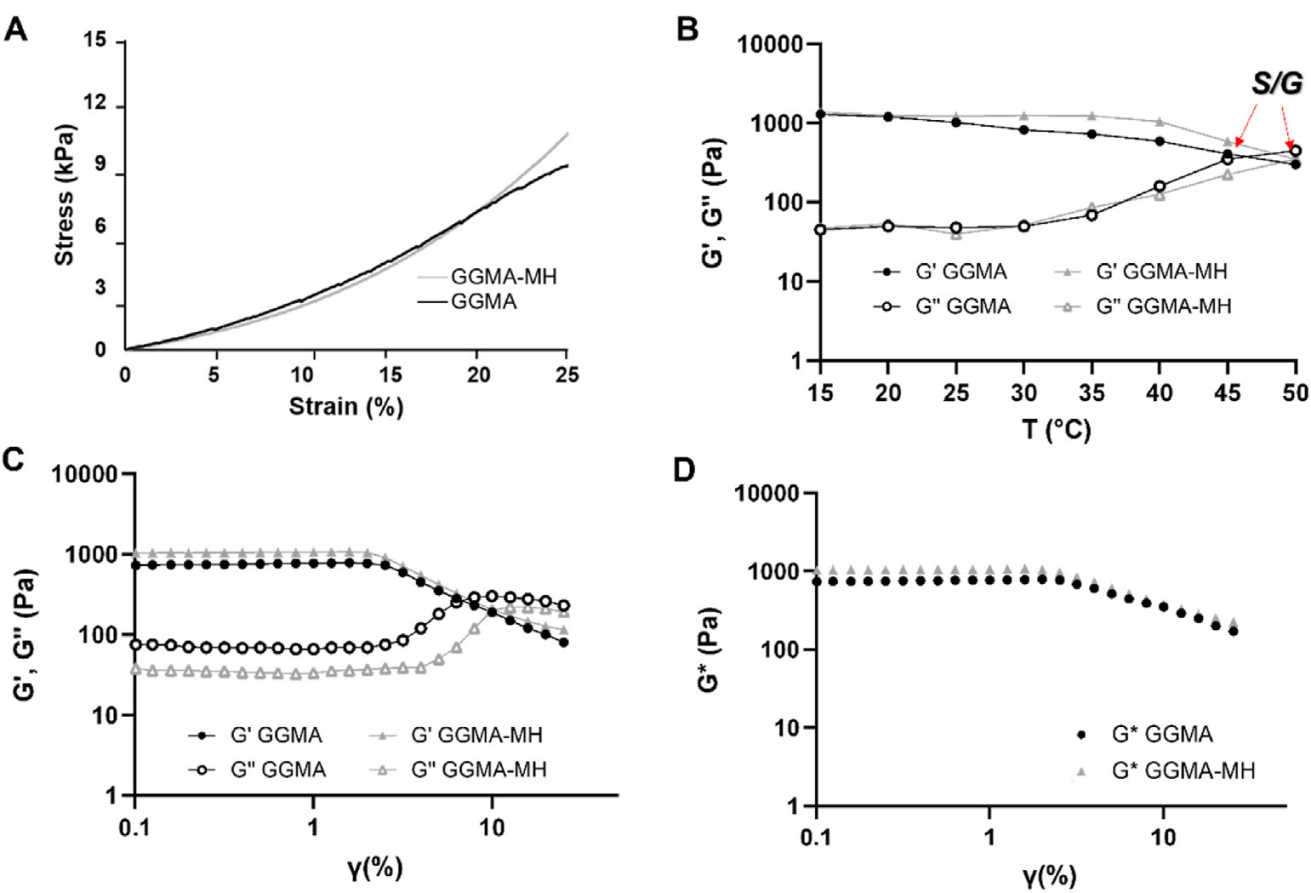
**(A)** Unconfined compression test for GGMA and GGMA-MH gels; **(B)** Rheological analyses for GGMA and GGMA-MH: temperature sweep test in the temperature range 15–45°C and record of G′ and G″ in LVER at each temperature – Red arrow are pointing at the Sol/Gel transition temperatures; **(C)** Strain sweep test at 37°C showing G′ and G″ with the increase of strain in the range 0.1–20%; **(D)** G∗ values with the increase of strain (0.1–20%). Tests were performed in triplicates.

A typical stress-strain (σ/*ε*) curve obtained from the unconfined compression test is reported in Fig. 3A for both GGMA and GGMA-MH hydrogels. It was calculated a compressive elastic Young's modulus from the linear part of the curve (0–10% strain) of approximately 26.3 ​± ​3.0 ​kPa for the GGMA gels and 23.6 ​± ​5.0 ​kPa for the GGMA-MH samples. After the linear region, it is recognised the densification region (more evidenced in the GGMA-MH sample), followed by the samples breaking, around 25–30% for the GGMA sample and 60% for GGMA-MH.

The temperature sweep test graph in Fig. 3B shows that G’>G″ at least of one order of magnitude for both GGMA and GGMA-MH, up to approximately 35°C; at 45°C it is visible the crossing point of G′ and G″ for the GGMA gel index of gel/sol transition. A similar tendency was observed for the GGMA-MH, achieving the crossing point at 50°C. The graphs of the strain sweep test (Fig. 3C and D), performed at 37°C, show the LVER of GGMA and GGMA-MH, whose strain limit was 8.0 ​± ​0.3% for the formed and 10.5 ​± ​0.2% for the latter. Both hydrogels showed G’>G” at least of one order of magnitude in the LVER. The calculated G∗ values in the LVER were 730.3 ​± ​50.2 ​Pa and 1042.1 ​± ​30.3 ​Pa for GGMA and GGMA-MH respectively. Also, the apparent viscosity (η) was evaluated, and the recorded values were 111.2 ​± ​20.0 ​Pa∙s for GGMA and 181.4 ​± ​10.6 ​Pa∙s for GGMA-MH.

### 3.2 Bioinks printing assessment and biological performances of the bioprinted constructs

**Fig. 4:**
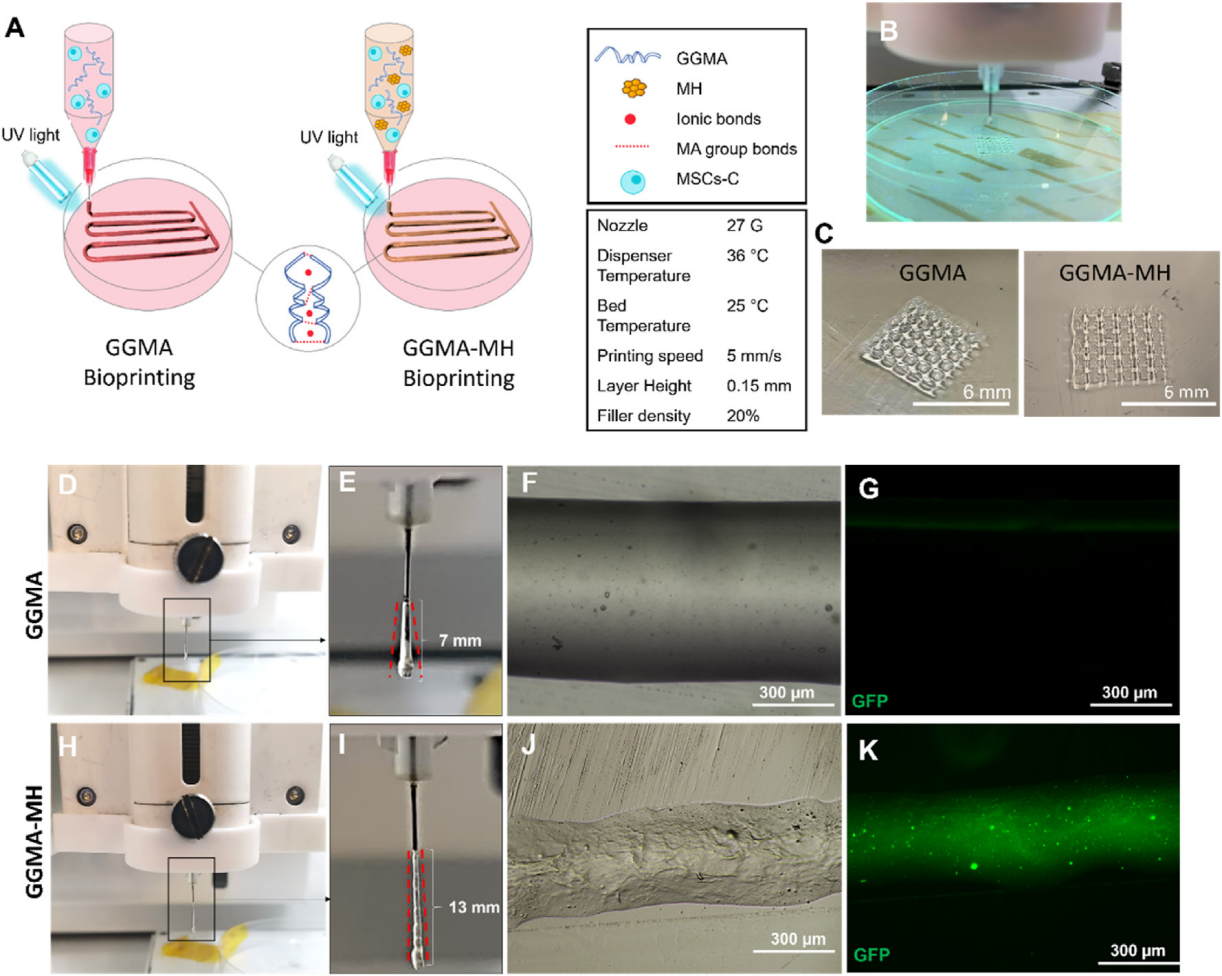
**(A)** Bioprinting process for MSCs-loaded GGMA and GGMA-MH bioinks via extrusion onto a DMEM/F12-covered printing bed: the obtained constructs were crosslinked via UV (MA groups bonds) and DMEM/F12 (ionic bonds); **(B)** Printing process with UV-light; **(C)** Bioprinted GGMA and GGMA-MH constructs; **(D,H)** Extrusion of GGMA and GGMA-MH formulations; **(E,I)** Zoom on the filament extruded before being deposited on the printing bed, red lines showing the change in the diameter over the filament length; **(F,J)** Phase contrast images of the extruded filaments of GGMA and GGMA-MH; **(G,K)** GFP images of the extruded filaments of GGMA and GGMA-MH.

For the printing process, the bioinks were prepared and loaded within the Rokit INVIVO biodispenser pre-heated at 36°C and the printing process parameters are reported in Fig. 4A. An example of the bioprinted GGMA and GGMA-MH constructs is reported in Fig. 4C and the video reproducing the printing process both during the printing of a single GGMA acellular construct and multiple cellular GGMA-MH construct are reported in the Supplementary Video and in Figure S3. The presence of MH led to a more stable and viscous filament extruded and therefore a better resolution. For the printability assessment, it was measured a spreading ratio of 5.3 ​± ​0.8 for the GGMA and 3.5 ​± ​0.1 for GGMA-MH. The GGMA-MH filament showed a more homogenous diameter distribution alongside the length compared to the GGMA, whose diameter increased from 0.7 ​mm at the top of the filament to 1.5 ​mm at the bottom. On the other side, GGMA-MH filament showed a diameter ranging from 0.5 ​mm when exiting the nozzle to 1.0 ​mm at the bottom. Also, the addition of MH helped to extrude a longer fibre (13 ​mm) compared to the bare GGMA whose filament resembled more a drop shape with a total length of 7 ​mm (Fig. 4D,E,H,I). Also, after the fiber deposition and UV-crosslinking, the filament diameter was measured (from images in Fig. 4F,J), reaching a value of ∼7 ​mm for the GGMA and ∼3 ​mm for the GGMA-MH. In Fig. 4G,K are reported the images of the filament in the GFP channel, to demonstrate the presence of MH which is auto fluorescent.

Supplementary video related to this article can be found at https://doi.org/10.1016/j.mtbio.2022.100287

The following is the supplementary data related to this article:

Video 1 2 Video 1

**Fig. 5:**
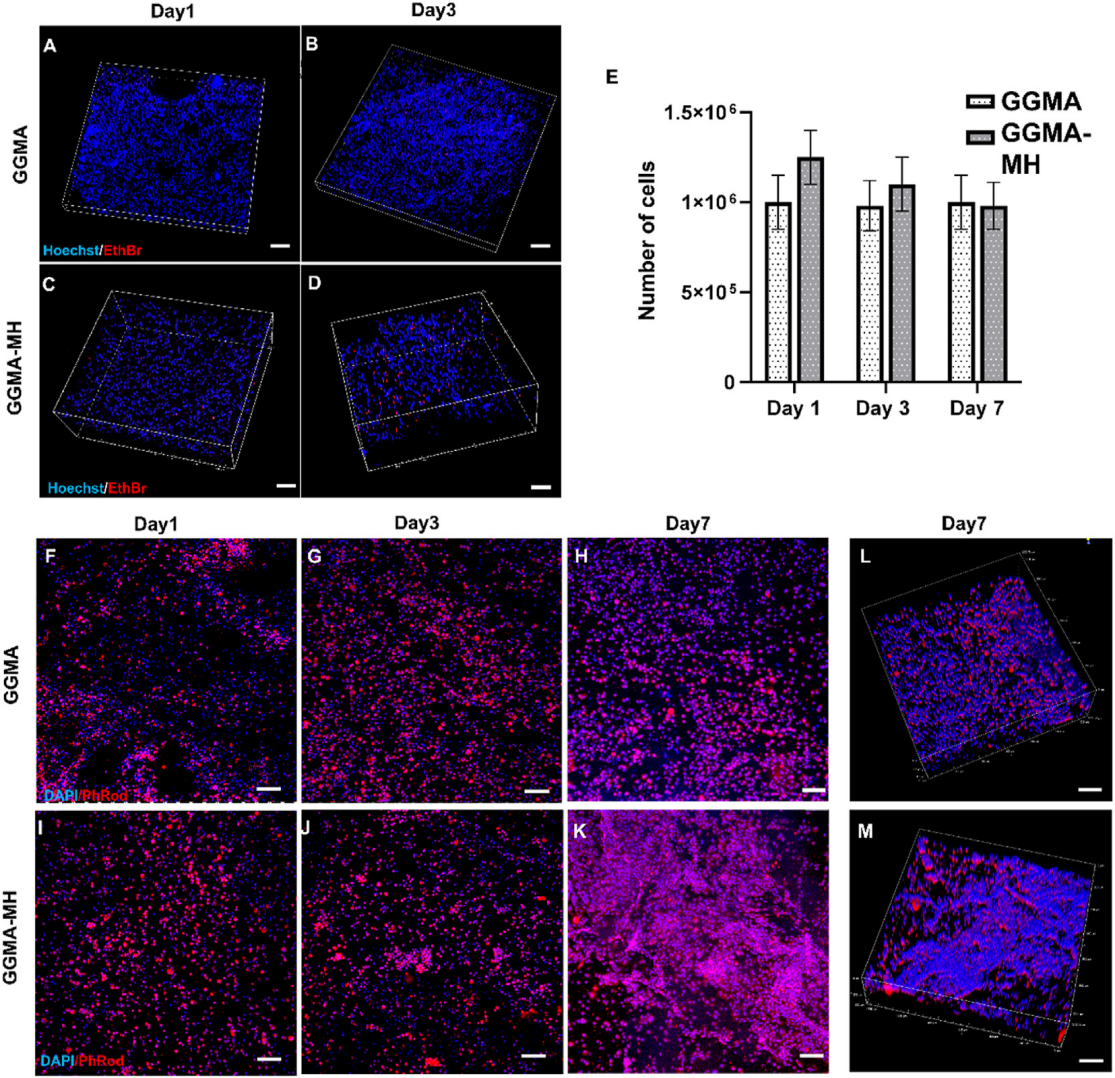
(**A–D**) Cytocompatibility evaluation of bioprinted construct via confocal microscope 3D images: ReadyProbes assay for MSCs-loaded GGMA and GGMA-MH bioprinted construct at day 1 and day 3 of culture (all the cells are in blue (Hoechst) and dead cells are in red (EthBr)); **(E)** Analysis of cells metabolic activity with MTS assay at day 1, day 3 and day 7. Tests were performed in triplicates. **(F–H)** Fluorescent labelling of bioprinted constructs staining Nuclei in blue (DAPI) and cytoskeleton in red (PhRhod): 2D images at days 1, 3 and 7 for GGMA and **(I–K)** GGMA-MH; 3D images at day 7 for **(L)** GGMA and **(M)** GGMA-MH. Bars ​= ​100 ​μm.

Fig. 5 shows the cytocompatibility results of the bioprinted constructs assessed on days 1 and 3 of cell culture. In the GGMA samples (Fig. 5A and B), the majority of cells were alive with a small percentage of dead cells on day 1 (2.0 ​± ​0.5%) and day 3 (1.8 ​± ​0.4%), while in the GGMA-MH samples (Fig. 5C and D) more red-stained cells were present on both days 1 (9.5 ​± ​3.5%) and 3 (18 ​± ​6.0%) (Graph of cells death percentage in Supplementary data). Also, cells exhibited a stable metabolic activity, evaluated with MTS up to 7 days of culture, without showing significant statistical differences neither between the two samples nor for the single samples within the increase of culture time (Fig. 5E). Furthermore, to reveal cell distribution within the bioprinted hydrogels on days 1, 3 and 7 of culture, fluorescent labelling was performed for both formulations (Fig. 5F–K). The rounded shape of MSCs-C cells was observed in both samples in 2D and also in 3D representations (Fig. 5L,M). Cell distribution within the construct was quite homogenous in GGMA samples over 7 days of culture, while it appeared to be more heterogeneous in the GGMA-MH samples on day 7, with the presence of cell aggregates.

**Fig. 6:**
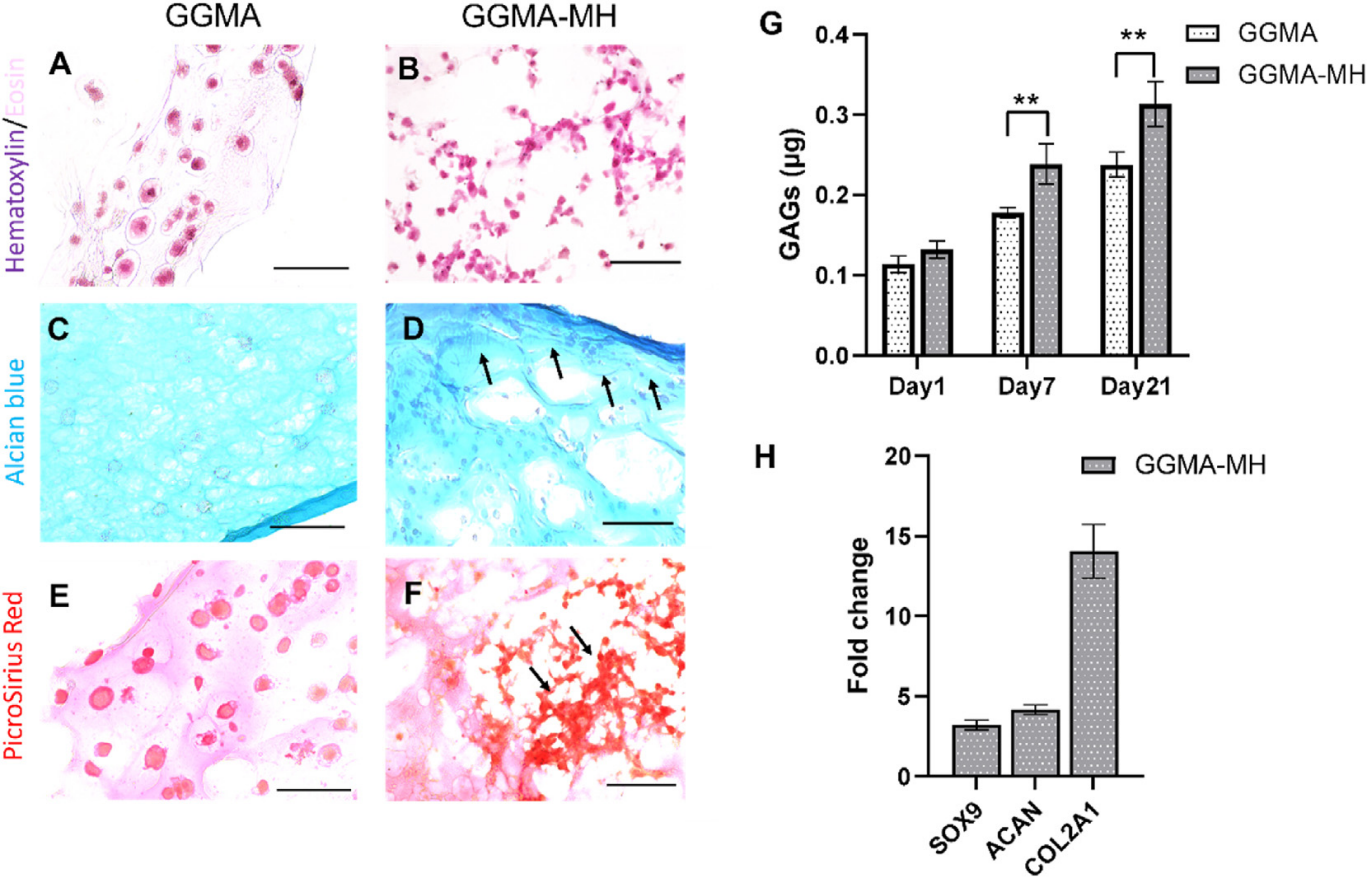
Histology staining of bioprinted constructs after 21 days of culture: H&E staining of **(A)** GGMA and **(B)** GGMA-MH; Alcian blue staining GAGs for **(C)** GGMA and **(D)** GGMA-MH; PicroSirius Red staining for **(E)** GGMA and **(F)** GGMA-MH. Arrows pointing at the high deposition of GAGs and Collagen. Bars ​= ​150 ​μm; **(G)** GAGs quantification at days 1, 7 and 21 of cell culture. There is a statistical difference between the three time points in each condition (∗∗∗∗p ​< ​0.0001); **(H)** Gene expression analysis for sox9, col2a1 and acan at day 21 for GGMA-MH (fold change with respect to GGMA). Statistics: ∗∗∗∗p ​< ​0.0001, ∗∗p ​< ​0.01. Tests were performed in triplicates.

H&E, Alcian Blue and Picrosirius red staining were performed for histological evaluation of the cartilaginous-like constructs (Fig. 6). After 21 days of GGMA and GGMA-MH bioprinted constructs culture, H&E staining showed many chondrocytes within the obtained constructs, mainly localised as typical isogenous groups, and with a relatively regular arrangement in the GGMA samples in comparison with the GGMA-MH, where cells appeared more agglomerated (Fig. 6A and B). Histochemical staining showed that the constructs both in the presence or absence of honey were strongly stained with Alcian Blue for GAGs, as well as with Picrosirius Red for Collagen (Fig. 6C–F). However, a greater amount of deposited matrix made of collagen and GAGs was observed in presence of Manuka Honey, as pointed out by the black arrows (Fig. 6D,F). From the GAGs quantitative analysis (Fig. 6G), both samples showed a significant (p ​< ​0.0001) increase in GAGs production on day 7 and 21 of culture, compared to day 1. In addition, GGMA-MH constructs exhibited a greater amount of produced GAGs on day 7 and day 21 compared to GGMA (p ​< ​0.01), suggesting a more gradual ECM formation. Gene expression analysis via RT-qPCR showed an increased expression of chondrogenic markers (i.e., *sox9*, *col2a*1 and *acan*) for GGMA-MH samples during the 21 days of culture compared to GGMA. Particularly, an increase of 3-fold, 14-fold and 4-fold was recorded for *sox9*, *col2a1* and *acan* respectively at day 21 in GGMA-MH, compared to bare GGMA (Fig. 6H).

**Fig. 7:**
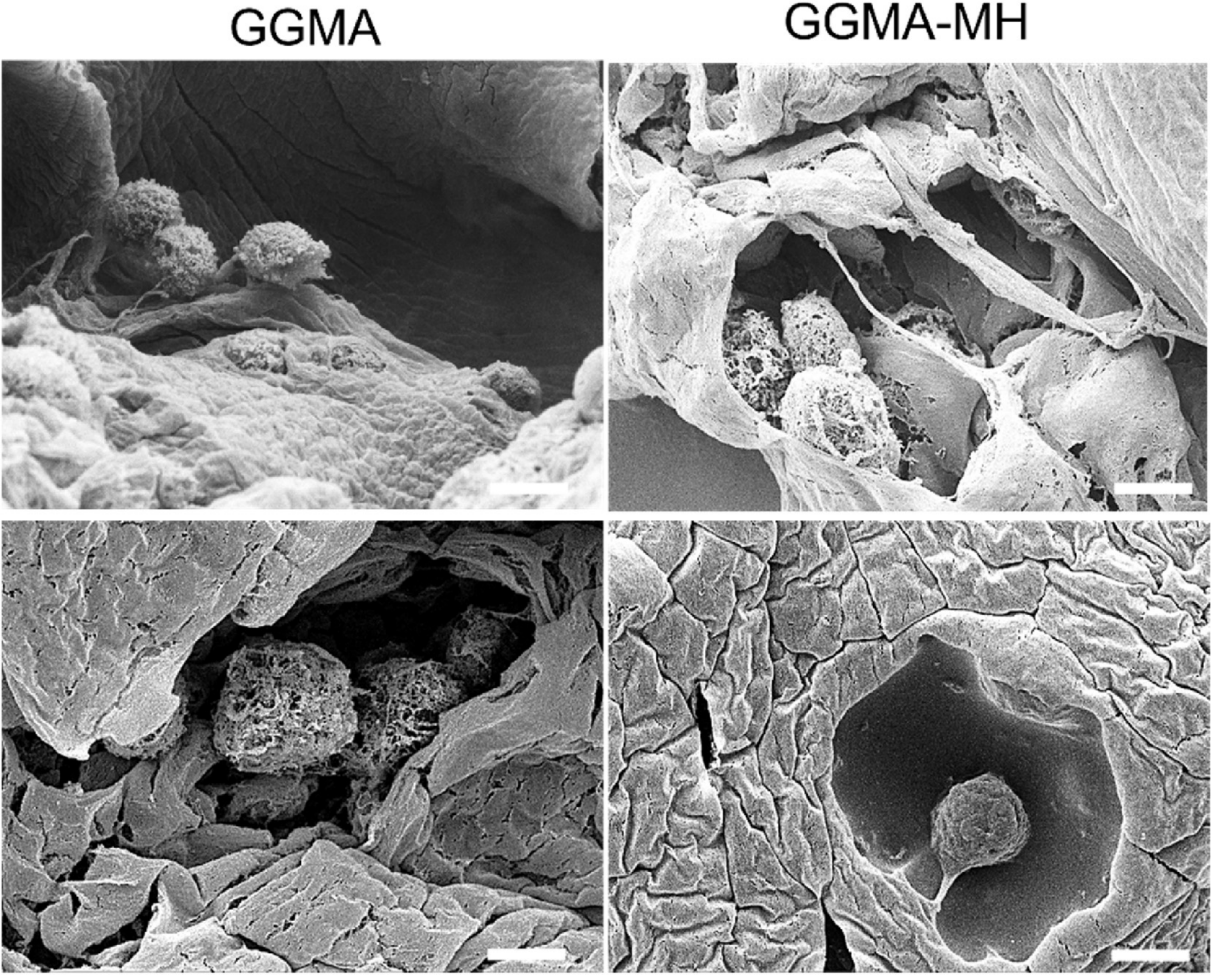
SEM micrographs of GGMA and GGMA-MH samples cross-sections at 21 days post-culture. Scale bars: 10 ​μm.

Finally, SEM was performed at 21 days on MSCs-C laden GGMA and GGMA-MH hydrogels to verify cell adhesion and cell-cell interactions in the network, as shown in Fig. 7. Both samples showed numerous cells dispersed within the matrix, possessing a round-shaped morphology with a diameter <20 ​μm.

## 4 Discussion

Although AC was predicted to be one of the first tissues to be successfully engineered, the current tissue engineering strategies are still unable to manufacture constructs indistinguishable from native cartilage [40]. To this aim, a technique tuneable with physical dimensions and properties is required; therefore, bioprinting technology provides the necessary capabilities [41]. In this work, we aimed to synthetize novel GG-based bioinks loaded with MH, chemically modified by the addition of methacrylic groups within the GG polymer chains, to obtain hydrogels chemically cross-linkable by photo-curing. Firstly, the methacrylation process has been analysed by different chemical techniques (Figure S1). FTIR-ATR spectra of the GGMA samples showed the appearance of the carbon double bond peak at 1640 ​cm^−1^, present in methacrylate groups but not in GG chains and the characteristic C=O peak of the ester bond appeared around 1700 ​cm^−1^ [41]. Also, the NMR spectroscopy confirmed the methacrylation of GG by the appearance of four characteristic peaks ^1^H MAS spectra corresponding to the presence of the mobile methacrylate in the sample. In addition, the heteronuclear ^1^H–^13^C correlation spectrum of methacrylate showed an extra peak at about 18 ​ppm for ^13^C and 1 ​ppm for ^1^H, showing that methacrylate molecules are close to gellan gum, and they are sufficiently rigid to cross polarize. Finally, XPS data showed a significant increase in the C/O area ratio on GGMA samples with respect to GG ones, thus suggesting the success of the methacrylation reaction. This increase can be related to the introduction of methacrylic moieties bringing four carbon atoms vs. one oxygen atom per grafted molecule. Moreover, after methacrylation, an evident decrease of C-OR/COOR and O–C–O/COOR area ratios was observed. This decrease was ascribable to the increment of COOR contribution due to the functionalization reaction.

Following, for the hydrogel preparation, the gellan gum concentration chosen was 2% w/v, since Coutinho et al. discovered that, when the concentration of GG polymer increases from 0.5% w/v to 2% w/v, there is a more intensive aggregation of the polymeric chains and a stronger physical and chemical crosslinking of the hydrogel formulation, without affecting the cytocompatibility of the biomaterials [42]. Also, GGMA 2% did not show detrimental effect on both cells’ viability on human bone marrow-derived MSCs and nasal chondrocytes [43]. Furthermore, in this work we decided to exploit a Nature-inspired strategy, as previously performed, adding the Manuka Honey to GGMA composition to assess two hypotheses: the ability of MH to enhance the GGMA viscosity, obtaining a more stable construct after bioprinting, and its effect on cells metabolism and cartilage production. MH is very attractive because of the already proven remarkable non-peroxide antibacterial activity of methylglyoxal (MGO, or Unique Manuka Factor) [34]. The MH concentration chosen was 5% w/v, as it provides minimal inhibition against different pathogenic bacteria [44] and, on the other side, was the lowest concentration able to affect the formulation viscosity, as demonstrated by the rheological analyses (Fig. 3). Besides, higher concentrations of honey could have a cytotoxic effect and its uncontrolled release over time could represent a hurdle in the development of honey-containing tissue-like substitutes [45]. Thus, for hydrogel manufacturing, two different compositions were exploited and compared: GGMA 2% w/v and GGMA-MH (2% w/v-5% w/v).

The gelation time (Fig. 1A) was assessed for both formulations and it was found to be 10 ​min when exposing the solutions at RT to UV lamp. Besides, the gelation of GG is strongly affected by the presence of specific cations, namely Na^+^, K^+^, Ca^2+^ and Mg^2+^, which could increase the double helix formation and the establishment of junction zones, leading to the formation of more crosslinked networks [46]. In presence of these ions, aqueous solutions of gellan gum undergo thermo-reversible gelation when cooled at RT. On the other side, divalent cations promote more efficient gelation than monovalent cations [29]. In this regard, we used a combination of photo-crosslinking (UV-curing) and ionic crosslinking, in response to temperature decrease and the presence of divalent cations, adding DMEM/F12 media to the solutions (which possesses Calcium Chloride (CaCl_2_), Magnesium Sulfate (MgSO_4_), Sodium Phosphate dibasic (Na_2_HPO_4_), Potassium Chloride (KCl) and Sodium Chloride (NaCl)). The double-crosslinking method decreased the gelation time to about 3 ​min, which represents a suitable time for bioprinting applications [47]. Furthermore, the effect of ionic solutions (made of monovalent ions) and the MH presence on the water-uptake kinetics was assessed by immersing the hydrogels in PBS solution for up to 48 ​h (Fig. 1C). Both GGMA and GGMA-MH hydrogels showed high hydrophilicity, with a fast WU increase when soaked within 1 ​h. Also, GGMA-MH samples showed excellent water absorption capacity, achieving swelling equilibrium after the rapid growth, within 1 ​h, demonstrating a high ability to retain water. Conversely, the GGMA samples reached a plateau only after 8 ​h of immersion. Besides, MH-loaded samples absorbed three-fold less water (1800 ​± ​20% GGMA vs 500 ​± ​60% GGMA-MH), due to the high amount of MH in the formulation, which implied a reduced hydrophilic (due to the presence of hydrophobic compound in MH) and crosslinked polymer content in the composites [48]. Therefore, the increase of material present in GGMA-MH provides less space for the overall water uptake, leading to a lower swelling ratio in the equilibrium state [49]. Conversely, both compositions showed a similar porosity as demonstrated by SEM analysis (Fig. 2A–D), with a porous interconnected structure, and pore size in the range 50–250 ​μm, optimal for nutrition supply and diffusion [50]. Interestingly, GGMA-MH hydrogel exhibited a more heterogeneous pores size, with diameters varying in a wider range (78–200 ​μm), compared to GGMA samples, where most of them were in the range 60–120 ​μm (Fig. 2E). 200 ​μm in pore size is considered optimal for hydrogels for cartilage regeneration, providing space for cell growth, adhesion, and proliferation of cells and ECM deposition [51]. To study the mechanical properties of the fabricated hydrogels, compression testing and rheological analyses were performed on GGMA and GGMA-MH hydrogels. In both cases, stable hydrogels were formed without significant differences in Young's modulus (E) of around 25 ​kPa, which is comparable to the values found in the literature for AC tissue engineering application of hydrogels [47]. Particularly, the values obtained would be optimal for mimicking the middle layer-cartilage, according to the studies performed before [52]. Remarkably, MH-samples presented longer stability up to 50% strain compared to the bare GGMA samples which broke at 30% strain, due to the higher flexibility and elasticity material, elasticity of the material, given by the presence of the honey [53]. This tendency was confirmed by rheological analyses (Fig. 3): the Strain sweep test demonstrated that MH-loaded samples possessed a higher stretchability, showing a strain value at the yield point of 10.5 ​± ​0.2% compared to the 8.0 ​± ​0.3% of bare GGMA samples. Also, the value of G∗ direct measure of the rigidity of a material's soft solid structure was higher in the case of GGMA-MH (1042.1 ​± ​30.3 ​Pa) compared to GGMA (730.3 ​± ​50.2) Pa and the apparent viscosity η, which was recorded as 111.2 ​± ​20.0 ​Pa⋅s for GGMA and 181.4 ​± ​10.6 ​Pa⋅s for GGMA-MH. These results confirmed the beneficial effect of Manuka Honey in improving the viscoelastic properties of the GGMA hydrogel composition.

These two sets of hydrogel formulation, in combination with MSCs differentiated into chondrocytes were used as bioinks for obtaining bioprinted cartilage-like constructs (Fig. 4). The concentration of cells chosen was 7x10^6^ ​cells/mL, as the optimal cell density for AC regeneration ranges between 5 and 20 million/mL. We noticed a higher printability of the GGMA-MH/MSCs-C bioink, in line with the rheological results obtained. Despite both samples demonstrated the successful printing process, with the obtainment of a stable multi-layered structure and a good shape fidelity, the addition of MH lead to a better printability. Indeed, MH-loaded bioinks displayed a spreading ratio of ∼3.5 compared to ∼5.3 of GGMA. Lower spreading ratios, approaching 1, which is the ideal ratio, are necessary to allow the fabrication of cell-laden hydrogel structures with high accuracy [54]. Also, it allowed the obtainment of a fiber-like filament two-fold longer than the drop-like filament obtained in absence of MH. This behaviour could be related to an increase in the shear-thinning performance of the GGMA formulation with the addition of MH, which allows the shape preservation of the bioink during the extrusion [[55], [56]]. This result, as anticipated, could be related to the viscosity of the Manuka Honey, which is a unique intrinsic property that already showed to enhance hydrogels’ mechanical features [57].

Concerning cell-laden bioprinting, obtaining favourable cell viability is critical for fabricating a biomimetic tissue construct. Many factors affect cell viability during bioprinting, such as bioink rheological properties (e.g. viscosity and shear-thinning property) as well as the crosslinking strategy (e.g. exposure time to UV light) and bioprinting parameters (temperature, printing speed) [56]. Regarding this, the dispenser temperature was set to 36°C, in order to not affect cell viability and to have a semi-liquid solution within the syringe before the extrusion. The stability of the strand after printing was guaranteed by the thermosensitive behaviour of the solutions, occurring due to the temperature decrease from the biodispenser (36 ​°C) to the printing bed (RT). The printing speed was set to 5 ​mm/s to get a fast process (around 2 ​min per construct) and to expose the cells to the UV light for a short time. Compared to the manual hydrogel polymerization for the GGMA and GGMA-MH fabrication, which requires 3 ​min of UV exposure and leads to the obtainment of a non-homogenous crosslinking of the whole construct, bioprinting reduced UV exposure to cells to 2 ​min for obtaining a crosslinked homogenous network, probably because of the gradual crosslinking during the layer's depositions [58]. Furthermore, no delamination was observed after one day, confirming a good adhesion of the printed layers and the stability of the 3D printed constructs in physiological environments. With GGMA-MH formulations, it was possible to print up to 10 layers without the structure collapse (Figure S4), but, in order to reduce the cells' exposure to UV light, a low-thickness construct was analysed (with just 4 layers).

As far as the biological performance of the bioprinted constructs is concerned, cell viability was confirmed on day 1 and 3 of culture, and metabolic activity was stable over 7 days of culture. The morphology and distribution of the cells within the constructs were different: in GGMA bioprinted construct, cells showed a homogenous distribution in the construct within the 7 days of culture, whereas in GGMA-MH sample cells were homogenously distributed on days 1 and 3, while they showed a tendency to form clusters at day 7, which was not surprising, considering the heterogeneity of pores dimension (Fig. 5) and the MH viscosity, leading to a non-uniform environment. Sridhar et al. demonstrated, via computational testing, that the optimal conditions for neo-tissue growth while maintaining structural integrity are met when cells are in dense and well-connected clusters [59]. In our work, H&E staining confirmed the tendency of MSCs-C to be more densely clustered at day 21 in GGMA-MH samples, confirming the behaviour shown by the Immunostaining analysis.

Also, histological analysis of collagen and proteoglycans at 21 days showed a greater amount of ECM produced by cells in GGMA-MH network, compared to GGMA bare samples (Fig. 6C–F). A similar finding was derived from the quantitative assessment of GAGs production on days 7 and 21, which displayed an increase of GAGs produced by cells over culture for both systems, with a significantly superior production in GGMA-MH constructs on day 7 and day 21 (Fig. 6G).

The transcription factor *sox9* is a master regulator of chondrogenesis and it is permanently expressed in adult chondrocytes where it manages AC maintenance and functions. *Sox9* can bind to the promoter of many genes expressed in chondrocytes including aggrecan (*Acan*); a*can* is a core linear peptide that binds several glycosaminoglycans chains and represents the most copious and biggest proteoglycan in the extracellular matrix; c*ol2a1* is the major component of the cartilage matrix [59]. The up-regulation of *sox9*, *acan* and *col2a1* expression at day 21 for GGMA-MH samples (with respect to GGMA calibrator), confirmed the chondrogenic fate of embedded cells and the positive effect of MH addition on cells chondrogenesis. Particularly, the increase of *col2a1* and *acan* expression is a marker of chondrocytes anabolic metabolism, proving that the GGMA-MH composition favoured ECM protein synthesis, as demonstrated by histological analysis and quantitative analysis of GAGs (Fig. 6H). [60]. While it has been widely reported that the antibacterial effect of MH is linked to the high level of methylglyoxal (MGO) formed from dihydroxyacetone (DHA), which is present in the nectar of the Manuka tree, other studies suggest that different active unknown honey components interact with the cellular pathways affecting, for example, cell proliferation or migration [61,62]. The greater chondrogenesis in MH-loaded samples could be related to the cells' agglomeration, which is an important feature characterising *in vivo* AC tissue development and formation: cell to cell contacts are established via cell surface molecules and when these contacts are formed, cells signalling activates the chondrogenic differentiation program which leads to the expression of cartilage markers such as collagen II and aggrecan [63,64]. Taken together, all these observations demonstrate that the presence of the MH, led to a more viscous and stable formulation, with better printability, and optimal cells distribution pattern, inducing the MSCs-C to an increase in ECM synthesis and chondrogenic potential (Fig. 7).

## 5 Conclusions

This research gave useful insights for future research on the specific component of the MH beneficial for chondrogenesis. However, this research field needs to be furtherly explored to understand cellular pathways and MH components cross-talk. The obtained construct could be therefore as a tool for a more in-depth study of MH effect on cells by genomic, immunohistochemistry and proteomic analysis, as well as used as a platform for future investigations on disease modelling and/or therapeutic treatments.

## Author contributions

Conceptualization: **A.S., A.M.F., E.D.G, P.G**.; writing—original draft preparation: **A.S., G.C., M.A.B., S·C.**; writing—review and editing: **A.S., X.N·W., M.M.B., E.D.G, P.G.**; data curation: **A.S., M.A.B., S·C., M.P., G.C.**; methodology: **A.S., S·C., M.A.B., S·C., X.N·W.**; formal analysis: **A.S., G.C., M.P., S·C., M.A.B., E.D.G., X.N·W.**; visualization: **A.S.; G.C., M.A.B**.; resources: **K.D., X.N·W., A.M.F., P.G**. supervision: **K.D., X.N·W., A.M.F., E.D.G., P.G**.

## Declaration of competing interest

The authors declare the following financial interests/personal relationships which may be considered as potential competing interests: Annachiara Scalzone reports financial support was provided by 10.13039/501100000266Engineering and Physical Sciences Research Council. Kenny Dalgarno, Xiao Nong Wang reports financial support was provided by 10.13039/501100012041Versus Arthritis.
