# Supplementary material for: Valuable effect of Manuka Honey in increasing the printability and chondrogenic potential of a naturally derived bioink
Source: Mater Today Bio. 2022 May 13;14:100287. doi: 10.1016/j.mtbio.2022.100287 (PMC9130107; doi:10.1016/j.mtbio.2022.100287)
Supplement: Multimedia component 1 [file mmc1.docx]

**Supplementary**

Synthesis of Gellan Gum Methacrylate (GGMA)

GGMA was synthesised by reacting Gellan Gum (Gelrite®, Molecular weight (Mw) = 1.000.000 Da) with methacrylic anhydride (MA) re-adapting a previous protocol by Coutinho et al (26). Briefly, 1 g of GG was dissolved in 100 mL of 1 M TRIS (Trizma® base) at pH 8.5-9.0, at 90 °C for 30 minutes. Then, MA (8% w/v) was added to the solution, when it reached 50 °C, to synthesize GGMA with a high degree of methacrylation (31). The reaction was continued for 5 hours, while the pH was monitored and continuously adjusted to pH 8 with the addition of 5 M NaOH dropwise. The obtained GGMA solution was purified by dialysis, using cellulose membrane with molecular weight cut-off of 11-14 kDa, for at least 3 days against distilled water (dH2O) to remove the un-reacted MA. Then, GGMA was stored at -20 °C overnight and then lyophilised for 48 h in a freeze-dryer (Alpha 1–2 LDplus, CHRIST, Germany) and stored in a vacuum chamber.

Characterization of GGMA

GG raw powder and freeze-dried GGMA were analysed with a Spectrum Two PE instrument equipped with a horizontal attenuated total reflectance (ATR) crystal (ZnSe) (PerkinElmer Inc., US). For both materials, the spectra were collected in absorbance mode, resulting from an average of 16 scans with 4 cm^−1^ resolutions. The wavelength range used for the recording was 4000–550 cm^−1^.

Nuclear Magnetic Resonance Spectroscopy (NMR) was performed on GG and GGMA samples to analyse the structure of organic compounds (Solid-State NMR Facility, Department of Physics, University of Warwick). The spectra were measured on a 4 mm HX probe in an 850 MHz spectrometer and the samples were spun at 12 kHz. For the ^13^C CPMAS experiments, 1024 scans were averaged for the GG sample and 4096 scans for GGMA. For the ^13^C{^1^H} CP hector experiments 8 scans were averaged for 96 increments for GG and 128 scans with 128 increments for GGMA. All ^13^C CP spectra were measured using 2 ms contact time, 3 s relaxation delay and 92.5 kHz 1H decoupling.

X-ray photoelectron spectroscopy (XPS) analyses were performed to gain insights into GG methacrylation reaction. A PHI 5000 VersaProbe II (Physical Electronics, USA) instrument, equipped with an AlKα X-ray radiation source, was exploited. Survey scans (0-1200 eV) and high-resolution signals in Fixed Analyzer Transmission mode (pass energy 29.35 eV), scanning areas of ~ 1400 x 200 μm were collected per dry sample. MultiPak software (v. 9.9.0.8) was used for data mining. Each peak area was normalised taking into account the sensitivity factors reported in the software library, thus the surface elemental composition was calculated. The lower binding energy of C1s photo-peak (e.g. C1s hydrocarbon peak) was set at 284.8 eV as charge reference.

Thermogravimetric analysis (TGA). TGA analysis was obtained heating 5-10 mg of samples in nitrogen-saturated atmosphere using PerkinElmer TGA-400 instrument (PerkinElmer Inc., Waltham, MA), operating within a temperature range 30-800 °C at a heating rate of 20 °C/min. The gas flow was set at 20 mL/min. Thermograms (TG) with respective derivative (DTG) curves were recorded and data were analysed using the software TGA Pyris series.

Count of dead cells

The percentage of dead cells within the GGMA and GGMA-MH bioprinted construct was analysed at day 1 and day 3. This evaluation was done by Image J on Image A-D of Figure 5.

Cell count were performed by ImageJ software (https://imagej.nih.gov/ij/) from three different images at 10 x magnification from each sample (experiments were carried out in triplicate).

**Results**

Synthesis and chemical characterisation of Methacrylated gellan gum

Figure S1A reports GG and GGMA FTIR-ATR spectra. The fundamental peaks of GG spectrum were: O-H stretching, observed at 3420 cm^−1^, the stretching vibration of aliphatic groups (-CH_2_ and -CH_3_), observed at 2970–2880 cm^−1^, the peaks associated with asymmetric (1618 cm^−1^) and symmetric (1412 cm^−1^) COO- stretching and the C-O stretching in the range of 1050 and 1000 cm^−1^ (35). Compared to GG, the GGMA spectrum exhibited a new peak typical of methacrylate group at 1645 cm^−1^ due to the addition of typical double bond (C=C) stretches, not well noticeable due to overlapping with the asymmetric COO− stretching band; a new peak related to the C-C stretching at 1536 cm^−1^ and the appearance of a peak at 1738 cm^−1^ indicating carbonyl stretching vibration of an ester (26). The insertion of methacrylate groups in the GG structure was validated by H-NMR analysis (Figure S1B). The chemical shift for unmodified GG presented characteristic signals at 5.15 and 1.32 ppm, corresponding to H^-1^ and H^-6^ of the α-anomers of L-rhamnopyranosyl residue. Additionally, the signals at 4.73 ppm and 4.55 ppm should be attributed to D-glucopyranosyl and D-glucuropyranosyl residues, respectively. These characteristic signals were also present in the chemical shift for GGMA. The GGMA spectra also showed the appearance of singlets at 1.96, 5.77 and 6.18 ppm which are ascribed respectively to the methyl proton of methacrylate and the two peaks from vinyl proton. Also, the heteronuclear ^1^H-^13^C correlation spectrum of methacrylate shows an extra peak at about 18 ppm for ^13^C and 1 ppm for ^1^H.

Further chemical characterisation was performed with XPS. GG and GGMA elemental compositions were studied, and the results are reported in Table S1. The main elements detected in both samples were carbon (C1s) and oxygen (O1s), while nitrogen (N1s) and sulphur (S2p) were present in smaller amounts. In addition, a significant increase in the C/O atomic area ratio (equal to 1.4 and 2.9, for GG and GGMA, respectively) was observed after the methacrylation. An accurate curve fitting of the C1s spectra (Figure S1C) revealed for GG samples five-components, as shown in Table S2. In GGMA samples, the same components were detected, but with different relative percentages. Indeed, in the GG sample the C-OR/COOR and O-C-O/COOR area ratios were 16:1 and 5:1, while in GGMA these ratios significantly decreased at 4:1 and 0.9:1.


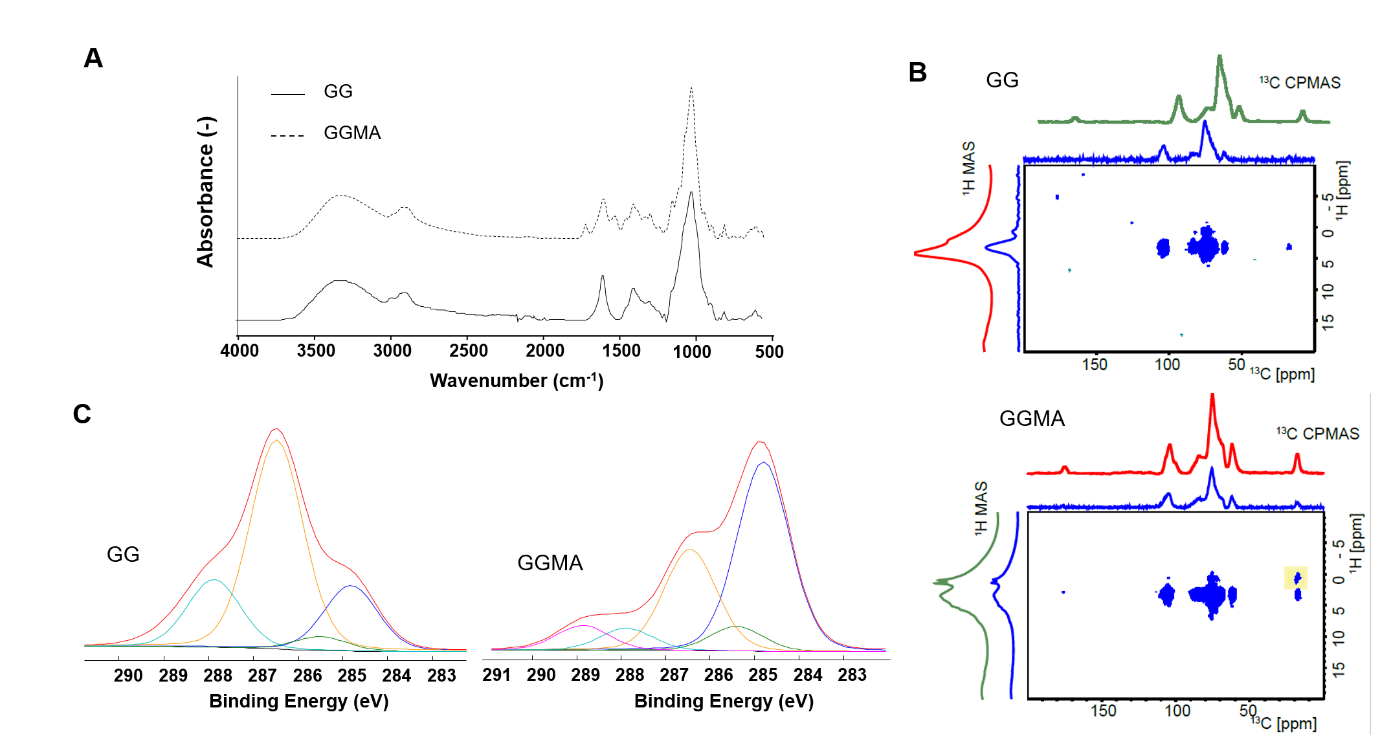


**Figure S1.** **(A)** FTIR-ATR spectra of low-acyl GG and GGMA freeze-dried samples; **(B)** NMR graph of GG before and after the methacrylation, relative to the 13C CP, 1H MAS and 2D 1H-13C heteronuclear correlation spectra with related ^1^H and ^13^C projections; **(C)** XPS data relevant to C1s curve fittings of GG and GGMA samples, whose attributions and atomic percentages were reported in Table 2.

**Table S1.** XPS Atomic percentage of each element for GG and GGMA samples.

|  | **Atomic %** | | | | | |
| --- | --- | --- | --- | --- | --- | --- |
| **Sample** | C1s | O1s | N1s | S2p | Na1s | Ca2p |
| **GG** | 56.9 | 40.1 | 1.9 | 0.5 | 0.3 | 0.3 |
| **GGMA** | 71.9 | 25.2 | 2.0 | 0.9 | - | - |

**Table S2.** XPS high resolution C1s and N1s peaks for GG and GGMA samples.

|  | **BE (eV)/ At%** | | | | | | | |
| --- | --- | --- | --- | --- | --- | --- | --- | --- |
| **Sample** | **CH_x_** | **C-COOR,**  **C-NH** | **C-OH,**  **CH-NH_3_^+^** | **O-C-O,**  **C-O-SO_3_^-^,**  **CO-NH** | **COOR** | **NH_2_** | **NH-C=O** | **NH_3_^+^** |
| **GG** | 284.8/  17.7% | 285.5/  3.6% | 286.5/  56.6% | 287.9/  18.4% | 288.8/  3.6% | 399.6/  35.2% | 400.1/  56.2% | 402.2/  8.6% |
| **GGMA** | 284.8/  52.6% | 285.4/  6.7% | 286.5/  27.9% | 287.9/  6.1% | 288.9/  6.7% | 399.7/  49.0% | 400.0/  28.2% | 401.9/  22.7% |

TGA showed a three-step characteristic thermogram in the GG sample (Figure S2), wherein the first stage of weight loss of about 13% occurred in the temperature range of 30–180 °C, which was attributed both to loss of moisture (non-bound water) and bound water contained in the sample. The major weight loss of 60% took place in the second step within the temperature range of 230–520 °C and it could be associated with the degradation of the polymeric chains, with a Tpeak =262.5 °C. Finally, about 16% weight loss occurred around 600 °C. The thermogram of GGMA showed different degradation steps. The initial 11% weight loss, which occurred in the range 30–120 °C, was attributed to the elimination of water adsorbed from the crosslinked polymer structure. Comparing the methacrylated and pure GG samples, a slight reduction of the mass loss associated with water removal and, more importantly, a narrow range of temperature associated with this event was observed. This could be related to the highly efficient drying procedure of GGMA. The major weight loss of about 65% took place in a second step, occurring within the temperature range of 120–530 °C, with the overlapping of two main peaks, i.e., the first at 232 °C and the second at 252 °C. Finally, the degradation step at the highest temperature, present in GG, disappeared.

**
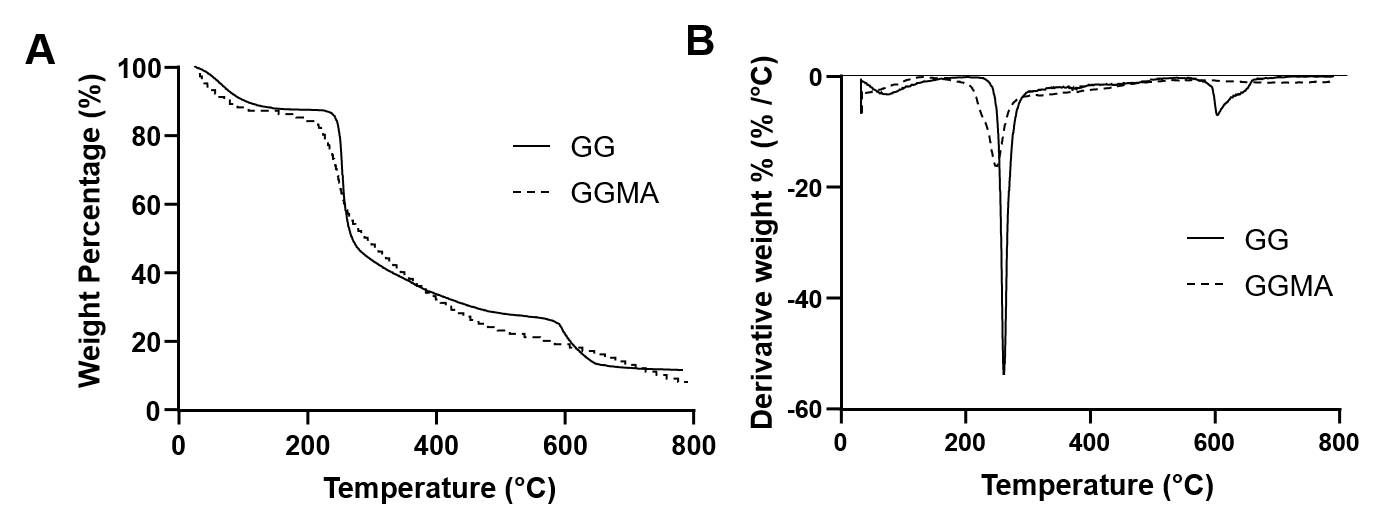
**

**Figure S2: (A)** Thermograms and **(B)** derivative thermograms relevant to GG and GGMA.

Bioprinting

An example of the GGMA-MH bioprinted construct made of 10 layers is reported in Figure S3, showing no collapse of the structure.


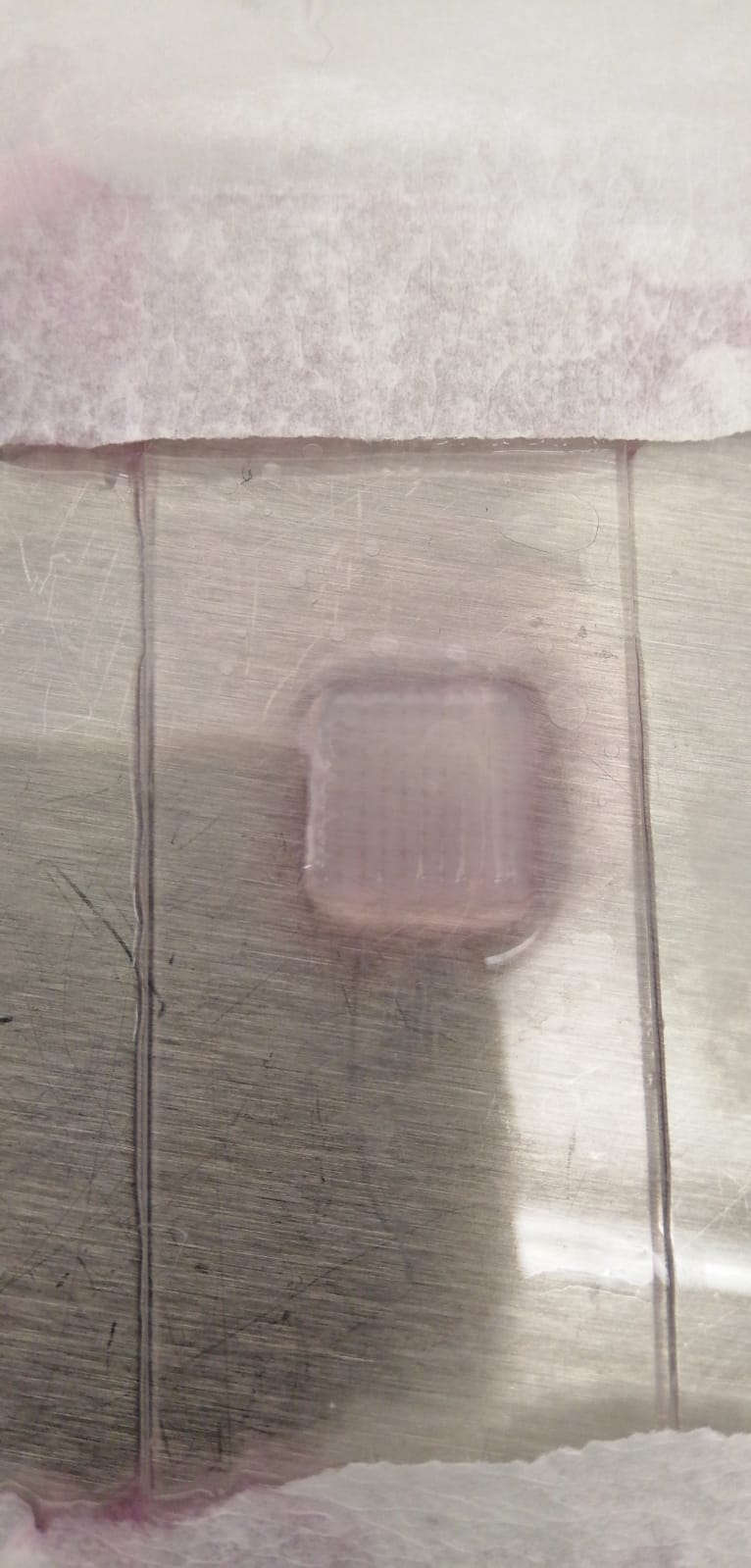


Figure S3: Example of GGMA-MH printability: 10-layers construct.

Count of dead cells

The percentage of dead cells within the GGMA and GGMA-MH bioprinted construct was found to be respectively: 2.0 ± 0.5 % at day 1 and 1.8 ± 0.4 % at day 3 for GGMA samples and 9.5 ± 3.5 % at day 1 and 18 ± 6.0 % at day 3 for GGMA-MH samples (Figure S4).

**Figure S4:** Percentage of dead cells at day and day 3 for GGMA and GGMA-MH constructs.
